# Mitochondrial ATP Synthase is a Target of Oxidative Stress in Neurodegenerative Diseases

**DOI:** 10.3389/fmolb.2022.854321

**Published:** 2022-02-14

**Authors:** Brad Ebanks, Lisa Chakrabarti

**Affiliations:** ^1^ School of Veterinary Medicine and Science, University of Nottingham, Nottingham, United Kingdom; ^2^ MRC Versus Arthritis Centre for Musculoskeletal Ageing Research, Nottingham, United Kingdom

**Keywords:** mitochondria, ATP synthase, oxidative stress, neurodegenarative disease, oxidative phoshorylation

## Abstract

The mitochondrial ATP synthase is responsible for the production of cellular ATP, and it does so by harnessing the membrane potential of the mitochondria that is produced by the sequential oxidation of select cellular metabolites. Since the structural features of ATP synthase were first resolved nearly three decades ago, significant progress has been made in understanding its role in health and disease. Mitochondrial dysfunction is common to neurodegeneration, with elevated oxidative stress a hallmark of this dysfunction. The patterns of this oxidative stress, including molecular targets and the form of oxidative modification, can vary widely. In this mini review we discuss the oxidative modifications of ATP synthase that have been observed in Alzheimer’s disease, Parkinson’s disease, and Huntington’s disease. Oxidative modifications of ATP synthase in Alzheimer’s disease are well-documented, and there is a growing body of knowledge on the subject in Parkinson’s disease. The consideration of ATP synthase as a pharmacological target in a variety of diseases underlines the importance of understanding these modifications, both as a potential target, and also as inhibitors of any pharmacological intervention.

## Introduction

### Neurodegenerative Diseases

Neurodegeneration is the process of progressive atrophy and loss of functional neurons in the central nervous system, and in particular the brain. The neurodegenerative process is not uniform, distinctions between which brain regions undergo atrophy, and by what molecular mechanisms the atrophy can be characterised, lead to the different neurodegenerative diseases that we now recognise (Damier et al., 1999; Sabuncu, 2011; Dugger and Dickson, 2017).

The most prevalent neurodegenerative disease is Alzheimer’s disease (AD), a form of dementia that is lived with by over 20 million people around the world, and in 2016 was the fifth largest cause of death at 2.4 million people (Nichols, 2019). As global population demographics shift towards older ages, the prevalence of the disease will increase, creating a huge health and social care as well as an economic burden (Castro et al., 2010; Marasco, 2020). The pathophysiology of AD is best characterised by three main characteristics: the aggregation of the amyloid beta (Aβ) protein into plaques (Selkoe and Hardy, 2016; Makin, 2018), the formation of neurofibrillary tangles by the tau protein (Braak and Braak, 1991; Gao et al., 2018), and the dysfunction of mitochondria (Moreira et al., 2006; Swerdlow, 2018), the cell’s bioenergetic and signalling hubs.

Parkinson’s disease (PD) is the second most prevalent neurodegenerative disease seen today. Characterised by classical motor symptoms of resting tremor and bradykinesia, much of this is due to the degeneration of the dopaminergic neurons of the substantia nigra (Hirsch et al., 1988). There are many genetic risk factors for PD, such as mutations to the *SNCA* gene that lead to the formation of α-synuclein aggregates known as Lewy bodies (Schulz-Schaeffer, 2010; Mahul-Mellier et al., 2020), as well as mutations to the *PINK1* and *parkin* genes that lead to failed mitophagy (the managed intracellular degradation of dysfunctional mitochondria), neuronal defects and even apoptosis (Goldberg et al., 2003; Palacino et al., 2004; Wood-Kaczmar et al., 2008).

Other less frequent forms of neurodegenerative disease include amyotrophic lateral sclerosis (ALS), ataxia, and Huntington’s disease. Huntington’s disease (HD) is caused by an excessive number of CAG repeats in the *Htt* gene, resulting in PolyQ repeats near the amino terminus. Accumulation of the mutant Huntingtin protein, as well as protein fragments, leads to the formation of toxic inclusion bodies that impact cell physiology, in particular proteostatic function (Bates et al., 2015). Mitochondrial dysfunction in the form of impaired complex II activity and decreased membrane potential have also been widely discussed in HD (Damiano et al., 2010). Atrophy of the medium spiny GABAergic neurons of the striatum is the classical neurodegenerative process observed in the disease (Rikani, 2014), and the major clinical presentations include involuntary movements and bradykinesia (Roos, 2010). With higher prevalence in people of European heritage, it is shown that around one in 7,300 from this demographic are affected (Bates et al., 2015).

### Redox Stress in Ageing and Neurodegenerative Disease

One common feature of neurodegenerative disease is the observation of high levels of oxidative stress within the cells of affected tissues (Lin and Beal, 2006; Smith and Cass, 2007; Sorolla, 2008; Wang, 2014; Liguori, 2018). This is in a large part due to the dysfunction of mitochondria that seems ubiquitous in these conditions. Mitochondria are the primary consumers of oxygen, they use molecular oxygen as the terminal electron acceptor at complex IV (cytochrome c oxidase) of the electron transport chain, reducing it to water (Kadenbach, 2021). This flow of electrons, provided by oxidised metabolites, facilitates the pumping of protons across the inner mitochondrial membrane and into the inter membrane space, generating a proton gradient. The activity of complexes I–IV of the electron transport chain is coupled to the activity of the enzyme ATP synthase, which harnesses the flow of protons from the inter membrane space back into the mitochondrial matrix to drive its rotary function, which ultimately provides the energy for the enzyme’s ATP synthesising catalytic activity (Gnaiger, 2020).

However, variations in this collective process leads to the production of oxygen based free radicals that are thought to be important signalling molecules (Zhang, 2016; Bárcena et al., 2018), but that can be harmful in excess (Perry et al., 1998; Redza-Dutordoir and Averill-Bates, 2016; Nissanka and Moraes, 2018). The endogenous production of free radicals largely occurs due to the leak or slip of electrons from complexes I and III, before their arrival at the catalytic site of complex IV, producing reactive oxygen species such as superoxide (O_2_^**.**^) (Chen et al., 2003; Balaban et al., 2005; Hirst et al., 2008).

### Forms of Protein Oxidation

The oxygen based free radicals such as superoxide and the hydroxyl radical, if not detoxified by antioxidants such as superoxide dismutase and catalase, go on to produce a variety of downstream radical species. In turn these species can modify the structure and function of proteins within the cell, and ultimately alter cellular physiology. While the radicals are capable of interacting with proteins directly (Stadtman and Levine, 2006), they are also known to lead to the modification of proteins through lipid peroxidation (Ayala et al., 2014), carbonylation (Fedorova et al., 2014), and also nitration (Ischiropoulos, 2009). These covalent modifications lead to often irreversible modifications to the proteins structure and therefore function, which often has detrimental outcomes. In order to determine the presence of these modifications, highly sensitive and specific assays can indicate the presence of a type of a modification within a sample (Shacter, 2000), while methods of liquid chromatography coupled with mass spectrometry can determine the presence of that modification to a specific protein (Hawkins and Davies, 2019).

### ATP Synthase Structure and Function

The ATP synthase enzyme itself is a large, two component enzyme that provides many targets for oxidative species to covalently modify. The insoluble F_O_ component is contained within the inner mitochondrial membrane and channels the protons from the intermembrane space to the mitochondrial matrix *via* its c-ring (composed of multiple subunit c peptides and subunit a) (Kühlbrandt and Davies, 2016). The movement of protons through the F_O_ component drives its rotary function, with the protruding central stalk (composed of the *γ*, *δ*, and *ε* subunits) stimulating conformational changes in the *α* and *β* subunits of the aqueous F_1_ component, which undertake the catalytic process of ATP synthesis from ADP and P_i_ (Abrahams et al., 1994; Noji et al., 1997; Stock et al., 1999; Von Ballmoos et al., 2009).

The aqueous component of ATP synthase is a target for the oxidative species produced by mitochondria, due to the proximity to the sites of radical production and the surface exposure of the aqueous F_1_ component. In one model of ageing the tryptophan-503 amino acid of the *α* subunit was identified as frequently oxidised in the *P. anserina* model of ageing (Rexroth, 2012). Reversible thiol oxidation of the α-subunit in the oocytes of *X. laevis* has also been observed to impact the function of the enzyme’s catalytic activity (Cobley et al., 2020). Other studies have also presented ATP synthase as a target of carbonylation and 4-hydroxy-2-nonenal (HNE) modification (Prokai et al., 2007; Jeong, 2008; Guo, 2011).

### Alzheimer’s Disease

Oxidative stress is widely observed in AD and is a defining characteristic of the mitochondrial hypothesis (Swerdlow, 2018; Butterfield and Halliwell, 2019). Within this hypothesis, it is described how oxidative stress is generated by intramitochondrial Aβ mediated dysfunction of the electron transport chain (Casley, 2002; Manczak, 2006). The original mitochondrial cascade hypothesis of AD describes how a gradual accumulation of oxidative damage to mitochondrial DNA, RNA, lipids, and proteins through dysfunctional electron transport chain activity contributes to disease pathology (Swerdlow and Khan, 2004). An updated consideration of this hypothesis considers how mitochondrial dysfunction can lead to oxidative stress, independent of intramitochondrial Aβ (Swerdlow, 2018). Aβ is also recognised as a source of neuroinflammation and oxidative stress in AD through its extracellular deposition in the brain that leads to the activation of microglia, the CNS macrophage (Colton et al., 2000; Koenigsknecht-Talboo and Landreth, 2005; Joshi, 2014; Zhong, 2018). Activated microglia contribute to the oxidative stress environment through the activity of the membrane-bound NADPH oxidase, and its release of the superoxide radical (Wilkinson and Landreth, 2006).

With mitochondria being a primary site of endogenous free-radical production and oxidative stress, the modification of ATP synthase by oxidative species is not unexpected. One of the first studies to identify the oxidation of ATP synthase in an AD context used *C. elegans* that overexpressed an aggregating form of GFP (as a proxy for protein aggregates observed in AD), and ATP synthase subunit *α* was identified as an oxidised protein (Boyd-Kimball, 2006). Since this preliminary observation, more detailed analyses of ATP synthase oxidation in AD have been produced.

Lipid peroxidation is a common hallmark of the oxidative stress that is observed in AD. The complex chemical interaction between ROS and polyunsaturated fatty acids leads to the production of reactive aldehydes, such as HNE, which can covalently modify proteins, modulating their structure and function. The first observation of HNE modified ATP synthase in the context of AD was from both the hippocampus and inferior parietal lobule (IPL) of mild cognitive impairment (MCI) patient brains, where ATP synthase subunit *α* was identified as excessively HNE modified (Reed, 2008). ATP synthase enzymatic activity was also reduced in both tissue types.

The Butterfield research group reported that the ATP synthase subunit *α* was again HNE modified in the IPL of early AD patients, considered to be the intermediate between MCI and AD (Reed et al., 2009a). ATP synthase was identified as HNE modified when the IPL of AD patients were studied, and this was again coupled with the observation that ATP synthase catalytic activity was reduced (Perluigi, 2009). A study of the entorhinal cortex from patients at Braak stages I/II observed the HNE oxidation of the ATP synthase subunit *α*, and the activity of the ATP synthase enzyme from the AD patient samples was found to be significantly decreased, while there was no observed decrease in activity of the major electron transport chain enzyme Complex I (Terni et al., 2010). At the earliest stages of AD, HNE modification of ATP synthase and subsequent downregulation of its activity has been observed. However, enzyme activity assays have suggested that this decrease in ATP synthase activity is not concordant with a decrease in Complex I activity, which suggests that a decrease in mitochondrial ATP output in early AD might be specifically dependent upon inhibited ATP synthase activity.

The frontal cortex of AD patient brains at Braak stages V/VI was assessed for a variety of oxidative protein modifications, and a four-fold increase in N^ε^-(malondialdehyde)-lysine (MDAL) modification of mitochondrial ATP synthase subunit *β* (Pamplona, 2005). However, when the entorhinal cortices of AD patients at Braak stages V/VI were assessed for variations in protein expression and for the presence of protein carbonylation, it was reported that while ATP synthase subunit β had upregulated protein expression, there were no significant differences in the carbonylation of the protein when compared with age-matched controls (Korolainen, 2006). When considered alongside the previously discussed studies that reported HNE modification of ATP synthase subunit α, this suggests that the oxidative modifications are specific to ATP synthase subunit α, and that changes in the oxidation state of the protein could be modified throughout disease progression.

Linked to the oxidative stress that characterises AD is the production of highly reactive peroxynitrite from nitrate and superoxide radicals (Radi, 2018). ATP synthase subunit *α* was identified as excessively nitrated in the hippocampus of AD patients (Sultana, 2006). When the IPL of early AD patients were measured, H^+^-transporting ATPase had excessive levels of nitration which was also coupled with a significantly reduced ATP synthase catalytic activity (Reed et al., 2009b).

**FIGURE 1:**
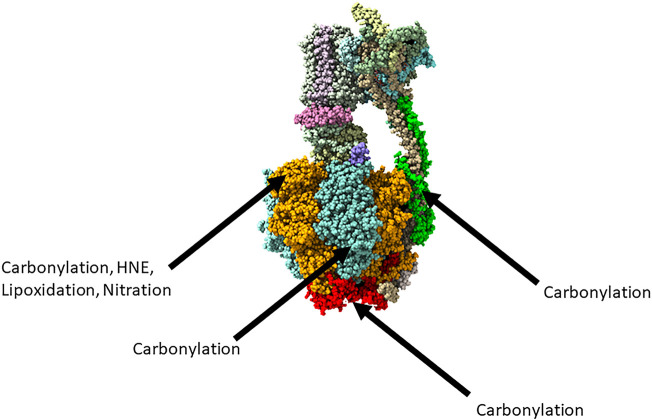
The reported oxidative modifications of ATP synthase in Alzheimer’s disease. Adapted from https://www.rcsb.org/structure/6ZPO. Subunit *α* represented in orange, subunit *β* represented in blue, subunit O represented in red, subunit D represented in green.

A study of the APP23 transgenic mouse model of AD in the pre-symptomatic stage considered the carbonylation of proteins that were extracted from cortex tissue (Hartl, 2012). The carbonylated forms of ATP synthase subunit *α*, ATP synthase subunit β, ATP synthase subunit b, ATP synthase subunit O, and the ADP/ATP translocase 1 were all more abundant in the APP23 transgenic mouse model than in the control. Primary cortical neurons of the APP23 mice were cultured and were found to have a significantly decreased ADP/ATP ratio than controls, pointing to a disrupted energy metabolism that appears at the very earliest stages of the disease. The observed oxidative modifications of ATP synthase in AD are summarised in Figure 1, adapted from Spikes et al. (2020).

### Huntington’s Disease

While oxidative stress is a common feature of both PD and HD, there are fewer observations of ATP synthase being a target of oxidative modifications in these diseases. However, a study of the oxidised proteins from the striatum of HD patients reported that ATP synthase subunit *α* is significantly carbonylated in comparison with age-matched controls (Sorolla, 2010). Enriched mitochondrial fractions were taken from the patient striatum samples and assessed ATP synthase catalytic activity, which was also significantly lower in comparison with controls.

### Parkinson’s Disease

Rotenone is a well-characterised inhibitor of the mitochondrial complex I that is used in studies of PD (Greenamyre et al., 2010). Complex I dysfunction is a major pathophysiological component of PD, and its inhibition by rotenone is utilised widely in both cell and animal disease models (KrakoJakovljevic, 2021). A study of dopaminergic PC12 cells exposed to rotenone observed that both ATP synthase subunit *α* and ATP synthase subunit β were carbonylated, to which the authors suggest could contribute to a reduced ATP output (Chiaradia, 2019). Mutations in the *SNCA* gene that encodes α-synuclein are also a feature of PD, as the aggregation of the protein leads to the formation of toxic oligomers and fibrils (Schulz-Schaeffer, 2010; Mahul-Mellier et al., 2020). Rat neuronal co-cultures treated with oligomers of α-synuclein showed a co-localisation of the oligomers with ATP synthase, which resulted in high levels of oxidised ATP synthase subunit *β* (Ludtmann, 2018). There is evidence of mitochondrial permeability transition pore (mPTP) opening in response to α-synuclein oligomer induced oxidative stress and compromised bioenergetics, and the role of ATP synthase as a structural component of mPTP is now increasingly better understood (Angeli, 2021).

*D. melanogaster* with *parkin* mutations are also a well-characterised disease model for PD (Greene, 2003). Studies have reported reduced Glutathione S-Transferase Omega (GSTO) in these mutants, which may result in increased susceptibility to oxidative stress due to the protective role of S-Glutathionylation against the irreversible oxidation of protein thiols (Song, 2014). When *parkin* mutants of *D. melanogaster* had GSTO1 restored, as it is downregulated in *parkin* mutants, the levels of S-Glutathionylation of ATP synthase subunit β increased, along with the catalytic activity of the ATP synthase enzyme (Kim et al., 2012).

## Conclusion and Perspectives

The mitochondrion is a primary site of free radical production in the cell, and thus a site of high levels of oxidative stress. This mitochondrial dysfunction that leads to oxidative stress has come to characterise many neurodegenerative diseases. The protein targets of this oxidative stress are varied but distinct to given diseases, and so in order to better understand the disease process, a comprehension of the oxidised proteins is required.

Proper functioning of the ATP synthase enzyme is essential to metazoan life, and in *H. sapiens*, its dysfunction is widely implicated in disease (Dautant, 2018). Here we have discussed the published evidence of ATP synthase modification by processes of oxidation in different neurodegenerative diseases, with a summary of these modification presented in Table 1. A more complete understanding of ATP synthase oxidation is presented in AD, largely based upon the work of the D. Allan Butterfield lab, but there is still more to be understood about this process.

**TABLE 1:** The different oxidative modifications of ATP synthase reported in Alzheimer’s disease, Parkinson’s disease, and Huntington’s disease.

Disease	Protein				
	ATP5A	ATP5B	Subunit d	Subunit O	ADP/ATP translocase
Alzheimer’s	Carbonylation, HNE, Lipoxidation, Nitration	Carbonylation	Carbonylation	Carbonylation	Carbonylation
Parkinson’s	Carbonylation	Carbonylation			
Huntington’s	Carbonylation				

Much less information is available in PD and HD but given that oxidative stress is a major feature in the pathophysiology of both diseases, effort should be directed toward understanding the oxidised proteome in both diseases, and in particular the oxidation of the ATP synthase enzyme. Other neurodegenerative conditions such not be overlooked in these investigations either, protein oxidation has long being understood to characterise ALS, but information regarding the specific proteins which are modified is lacking (Beal, 1997; Andrus et al., 1998).

Given the progression of the candidate drug J147, which targets ATP synthase, to clinical trial as a potential treatment for AD (Goldberg, 2018), as well as the deeper consideration of the enzyme as a target in anti-cancer therapies (Wang et al., 2021), a deeper understanding of the frequency, type, and location of the oxidative modifications of the mitochondrial ATP synthase enzyme should allow for the development of candidate drugs to treat these diseases in the future.
